# “Meet the patient” session: a strategy to teach medical students about autonomic dysfunction after spinal cord injury

**DOI:** 10.1186/s12909-023-04871-4

**Published:** 2023-11-23

**Authors:** Juliana Cazzaniga, Susan Solman, Jenny Fortun

**Affiliations:** 1 Herbert Wertheim College of Medicine, Florida International University, Miami, FL United States; 2 Department of Medical Education, Herbert Wertheim College of Medicine, Florida International University, Miami, FL United States

**Keywords:** Spinal cord injury, Autonomic dysreflexia, Autonomic dysfunction, Medical education, Patient panel, Neurological emergencies

## Abstract

Dysregulation of the autonomic nervous system is an important long-term consequence of spinal cord injury (SCI). Yet, there is a scarcity of teaching resources about this topic for preclinical medical students. Given the association of SCI sequelae with emergency complications and mortality, it is imperative to equip medical students with the ability to recognize them. We designed a “Meet the Patient” (MTP) session with the primary goal to enhance student learning about SCI sequelae by interacting with patients and listening to real-life stories. The session primarily focused on recognizing triggers and symptoms of autonomic dysreflexia (AD) and discussing the loss of bowel and bladder control, while providing opportunities to learn more about living with SCI from patients’ real-life experiences. During the MTP session, patients living with SCI discussed their experience with AD, neurogenic bowel and bladder, and spasticity, among other SCI sequelae. We evaluated the outcomes of the MTP session by assessing numerical performance in questions related to the session (post-session quiz and final exam) and students’ satisfaction (post-session survey) in two subsequent academic years. The numerical performance in SCI-questions was high for both academic years (and higher than national average for the final exam question), indicating adequate acquisition of knowledge. Satisfaction with the session was high, with most students indicating that the session helped them consolidate their knowledge about the topic.

## Introduction

Spinal cord injury (SCI) affects 1.3 million North Americans and has a significant impact on patients and society [1]. In addition to loss of motor and sensory function, SCI results in autonomic dysregulation, which influence the quality of life of patients and may lead to severe complications. An important example of this is autonomic dysreflexia (AD), a potential life-threatening emergency characterized by a sudden rise in blood pressure co-occurring with possible headaches, changes in heart rate, and profuse diaphoresis, among other symptoms [2, 3]. Although ~ 90% of patients with SCI above T6 experience AD, this sequela often goes unrecognized in emergency rooms and is a significant cause of morbidity and mortality [4]. Other long-term sequelae associated with autonomic dysregulation include loss of bowel and bladder control, thermodysregulation, systemic hypotension, sexual dysfunction, cardiac dysrhythmia and bronchoconstriction, among others [2]. Given that these complications are common and often associated with emergency situations, it is imperative to equip medical students with the tools necessary to recognize autonomic dysfunction after SCI, in particular AD.

Medical education faces the challenge of delivering a wealth of complex knowledge in a short amount of time [5]. Neurology is particularly challenging, with a well-described phenomenon of fear of neural sciences and clinical neurology among medical students (“neurophobia”) [6]. One strategy to combat neurophobia is the incorporation of active learning pedagogies, such as team-based learning, case-based learning, flipped classroom (FC) and simulations [5, 7]. Several studies have reported an improvement in knowledge acquisition and student perception with active learning, compared to traditional lectures [8–12]. In addition, these activities promote student engagement, participation and collaboration, and the development of skills that can be transferred to patient care [8, 9]. There has been an increase in the use of standardized patients and simulations to expose students to specific clinical conditions while teaching foundational sciences in preclinical years [13–15]. These sessions provide students with the confidence to incorporate knowledge into patient care and improve their communication skills [13, 16]. Moreover, they provide opportunities for students to develop and reinforce the necessary skills to interact with vulnerable populations [17].

Despite the impact of autonomic dysfunction, SCI teaching in medical education concentrates primarily on neuroanatomy [2, 18]. To our knowledge, there is no published active learning resource to teach autonomic dysfunction to first- or second-year medical students. Although there are reports of using standardized patients to teach AD to medical residents, there is a scarcity of these tools for undergraduate medical education [19–21]. Moreover, most of the available resources focus on multimedia, simulations, and standardized patients, but not actual patients or patient panels. The incorporation of real-life patients sharing their experiences and/or acting as “patient-teachers” may provide a more realistic experience and increase student level of comfort with the field and interest in neurology [22, 24].

In this paper, we describe the use of a novel session called “Meet the Patient” (MTP). In this session, patients living with SCI were brought to the classroom to talk to second-year medical students about their stories, emphasizing their experience with AD and loss of bowel and bladder control. Our major goal was for the patients to share their real-life examples of AD triggers, describe emergency situations where their AD was not recognized promptly in the hospital setting, their lifestyle adaptations after SCI concentrating on tools to manage their bladder and bowel, as well as some emergency situations they have experienced because of gastrointestinal or genitourinary dysfunction. Spasticity and clonus were demonstrated in class to provide some field exposure before clerkships. Importantly, we built within the session opportunities to enable students to practice communication skills, teamworking, and problem-solving to reinforce concepts learned in prior classes and make personal connections with patients.

## Methods

### Groups

The MTP session was delivered to second-year students enrolled in the “Nervous System and Behavior course”. We have conducted the MTP session in two subsequent academic years (AY1, 2021–2022, n = 108 students; AY2, 2022–2023, n = 133 students). Attendance was mandatory for AY1, but optional for AY2. Moreover, AY1 had 3 patients (2 h session) and AY2 had 2 patients (1 h session). The changes in AY2 regarding attendance requirements and the time of the session were made based on student feedback from AY1. In both years, one of the patients was a faculty member who helped facilitate the session, providing important teaching points in addition to giving insight as a patient (T3 injury). The other patients were different among the two cohorts, but they all have cervical injuries (different levels; C6 and C7 for AY1, and C4 for AY2). All patients were recruited based on their intrinsic motivations to contribute to SCI education and society; they had participated in a session about disability in the Clinical Skills course, except one patient who was recruited based on her connections with the SCI community. Patients not returning in AY2 were due to scheduling conflicts and difficulty traveling. The second facilitator for both years was a course core faculty member. The study received IRB Exemption approval (IRB-22-0472). All methods were carried out in accordance with relevant guidelines and regulations.

### Faculty and patient preparation

The faculty first met to design the session and discuss objectives and goals. Participating faculty met with the patients twice. In the first preparatory session, the format, goals, expectations, and other logistic details were discussed. Patients were briefed on the developmental stage of the student, and what they were expected to know (or not) and get out of the session. The questions were selected based on the objectives of the session and the topics that the patients felt comfortable discussing. Patients were given autonomy to select topics they thought were important to include. During the second meeting, faculty and patients went through a mock session to rehearse their answers and make them aware of the points at which the faculty member may interject to ask questions to the students. The primary role of the recruits was “patient teacher”, whereby they had a moderate level of autonomy and preparation for a specific teaching role about AD and were encouraged to have an interactive conversation with students, asking and answering questions about their experiences and understanding of AD [24].

### Logistics

The MTP session occurred immediately after an FC on spinal cord disorders (vascular, inflammatory, nutritional, infectious, and traumatic) that purposely omitted traumatic SCI and autonomic dysfunction. The FC followed a case-based approach whereby students were prompted to work in groups to localize spinal cord lesions, discuss specific disorders, and correlate neuroanatomy with functional loss. This was included before the MTP to strengthen students’ knowledge about the spinal cord. The MTP aimed to discuss the consequences of SCI, particularly as it relates to loss of autonomic function, focusing on AD and the loss of bowel and bladder control (topics not discussed in the FC). In addition, loss of motor and sensory function was reviewed, and clonus and atrophy were demonstrated in class. Students were given a faculty-developed study guide and a voice-over presentation to prepare for both sessions, FC and MTP, which included a description of the consequences of SCI and an explanation of the pathophysiology of AD. In addition, students had had prior lectures on the anatomy and function of motor and sensory tracts and autonomic systems.

During the MTP session, the faculty first introduced the activity and the patients. Initially, patients provided general information not related to the injury. Afterward, through a series of questions, they showed the students the movements they were able (or not) to do, as well as where they had (or not) sensations. They were told to not reveal the level of injury. Next, students were asked if they have more questions for the patients, after which, they were prompted to predict approximately what the level of injury was. A demonstration of spasticity and atrophy occurred at this point. After revealing their level of injury, the faculty asked previously rehearsed questions to the patients aimed at describing their experience with AD, and bowel and bladder control, including emergency complications. Students were encouraged to ask questions throughout the session. A second set of questions was prepared related to changes in lifestyle, thermodysregulation, sexual dysfunctions, nutrition, the use of assisted devices, etc.

### Evaluation

After the session, students were provided 24 h to complete a post-session on-line quiz on their learning platform (CanvasMed). The quiz was timed (90 s per question) and individual. There were 7-content related questions (graded): 3 about autonomic dysreflexia, and 4 related to signs and symptoms based on the level of injury. There were 5 additional questions (non-graded) about their satisfaction with their session at the end of the quiz (same entry in the online learning platform). Using a Likert-scale, students were asked specific questions about their satisfaction with the question overall and the impact of the session on their knowledge. The results from the post-session quiz were collected from CanvasMed, including difficulty index and point biserial. A two-tailed paired t-test was used to compare results in graded questions and satisfaction rate between academic years. In addition, a question about the session (autonomic dysreflexia) was included in their final exam from the National Board of Medical Examiner (NBME). The p-value and discrimination index, as well as the national p-value were provided by the item analysis from NBME.

## Results

The MTP session involved recruiting and training patients living with SCI and establishing a partnership between patients and faculty. Table 1 summarizes practical considerations for the inclusion of patients in this session.

**Table 1:** Practical consideration of patient inclusion in the MTP session

Points	Patients in our study
Patient recruitment and compensation	Have participated in activities at the school. Have personal connections with facilitators. Have intrinsic motivation and commitment to teach medical students. Were financially compensated.
Patient preparation	Held two meetings with facilitators: one for planning and one for rehearsing. Were briefed on learning objectives, level of knowledge of students, expectations from the session.
Patient role	Were encouraged to add topics for discussion. Were asked about comfort level or concerns discussing certain topics.
Patient feedback	Were asked for their feedback after the session and their views on the impact of the session.

**Fig. 1:**
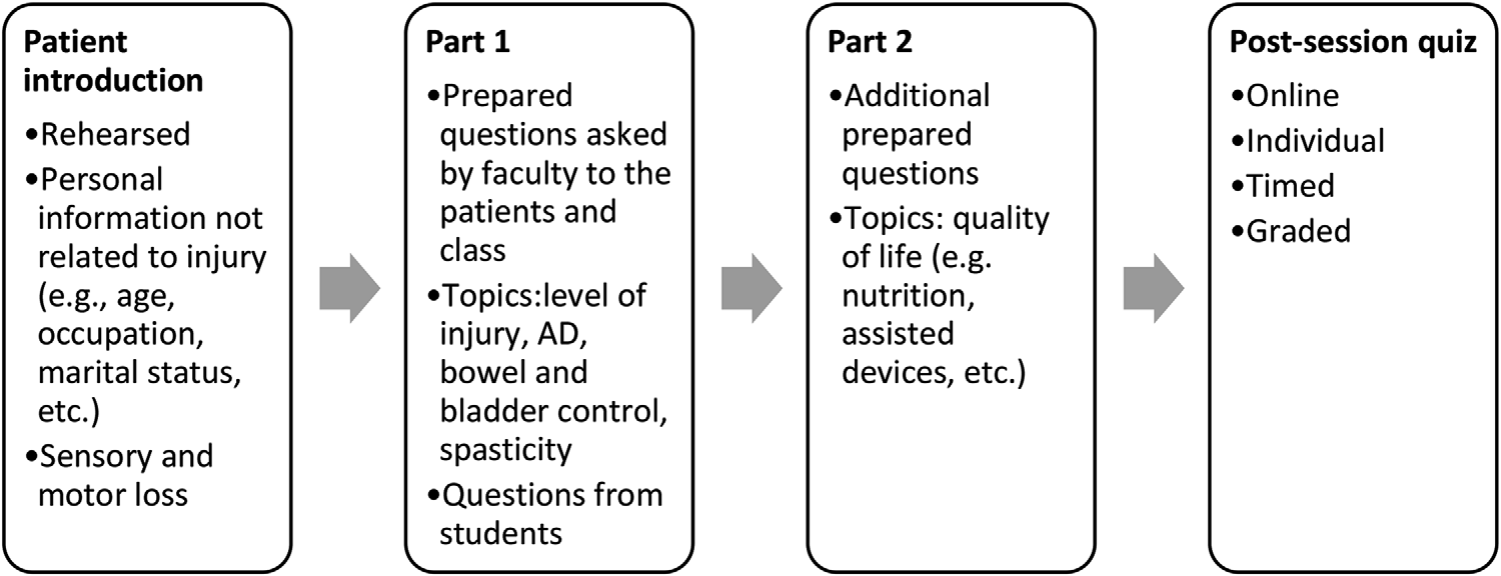
Diagram illustrating the progressive format of the MTP session in class

Attendance was 100% (mandatory) and 63% (optional) in the first and second years, respectively. We observed students paying attention, asking relevant questions to the patients, and participating in the discussion throughout the session. For both cohorts, in addition to motor and sensory loss specific to each injury, topics discussed included triggers for AD, symptoms exhibited, experiences in the emergency department, bladder and bowel routine, use of assisted devices for voiding, past surgeries and infections. Moreover, increased muscle tone, atrophy due to disuse, and clonus were demonstrated in the classroom. Additional topics discussed in the first session included nutrition (e.g., foods that aggravated constipation), catheterization, pressure injuries, neuropathic pain, pain management, thermodysregulation, and emotional and sexual relationships. Emphasis was made on the use of proper language when dealing with disabilities. Of note, topics such as management of emergency complications were not covered, as this is an objective of the clerkship phase of our curriculum (and not preclinical). Figure 1 depicts the format of the session.

Satisfaction with the session was overall positive in both academic years (Table 2). Most students agreed or strongly agreed that the MTP session contributed to their knowledge of disabilities related to SCI and AD (95% and 94% in AY1, 78% and 77% in AY2) and that the session was a worthwhile use of their time (95% and 76%). Fewer students, but still the majority, agreed or strongly agreed that the MTP session contributed to their knowledge of spasticity (81% and 73%) and bladder and bowel control (88% and 68%). The satisfaction rates were smaller overall in AY2, compared to AY1 (74.4 vs. 90.4, p < 0.05). To our surprise, most students in AY2 completed the on-line 5-questions satisfaction survey at the end of the graded post-session quiz, even though they did not attend the session or had the opportunity to watch a recording. A subgroup of students approached the patients after the session to express their gratitude (not prompted by us and not quantifiable).

**Table 2:** Student satisfaction at the end of the MTP session

Statement	AY	Strongly agree	Agree	Neutral	Disagree	Strongly disagree
The MTP session contributed to my knowledge of disabilities related to SCI.	1	69	26	5	0	0
	2	40	38	20	0	1
The MTP session contributed to my knowledge of AD	1	67	27	6	1	0
	2	35	42	20	1	1
The MTP session contributed to my understanding of spasticity.	1	54	27	14	6	0
	2	32	41	24	0	1
The MTP session contributed to my understanding of bladder and bowel control after SCI.	1	59	29	10	2	0
	2	30	38	27	2	1
The MTP session was a worthwhile use of my time.	1	68	27	6	0	0
	2	38	38	22	0	1

To evaluate knowledge acquisition, we administered a knowledge-based quiz after the session. The performance in the graded post-session quiz was similar between the two academic cohorts (average 0.90 and 0.89 in AY1 and AY2, respectively; p = 0.921; Table 3). In both years, the question with the highest level of difficulty was the same (identifying level of cervical injury). The final exam contained a question about AD. Class performance was similar in both years and 14-points higher than the national average in the same question (p value of 0.96 and 0.99 for AY1 and AY2, respectively, compared to a national average of 0.82, discrimination index 0.2).

**Table 3:** Student performance in the post- session quiz

		AY 1		AY2	
Question	Tag	Difficulty Index	Point Biserial	Difficulty Index	Point Biserial
1	AD- level	0.98	0.24	0.99	0.14
2	AD- symptom	0.99	0.041	1	0
6	AD- triggers	0.99	0	1	0
3	Level of injury-breathing	1	0	0.98	0.28
4	Level of injury-lumbar	0.92	0.37	0.91	0.63
5	Level of injury- cervical	0.50	0.69	0.64	0.62
7	Level of injury- thoracic	0.90	0.40	0.71	0.53

## Discussion

The field of neurology education has experienced significant changes that parallels the advances in technology and a growing understanding of both, the science of learning and neurology [22]. Given that autonomic dysfunction, including AD, is associated with potentially life-threatening complications, it is important to include this topic early in medical education and equip students with the skills needed to recognize it [2–4]. Despite several reports describing the use of patients in undergraduate medical education, we did not find examples of sessions involving patients living with SCI. Moreover, none of the resources we find in the literature to teach about AD involve patients that have real-life experience with it [19–21]. We developed and implemented a MTP session in which patients living with SCI shared their experiences with second-year medical students to complement the learning occurring in the course. Our goal was to foster not only knowledge but the humanistic and emotional aspects of medicine.

The importance of neurology field exposure in medical education depends on students being able to develop the necessary patient-centered skills to communicate and form doctor-patient relationships with a wide range of patients [23]. In our MTP session, students had many opportunities to interact with the patients, which allowed them to reinforce concepts learned, including identifying the level of injury, spasticity, signs and triggers of AD, and loss of bowel and bladder control, while reminding them why what they are learning is important. Similar to experiential learning theories, the MTP session emphasizes learning through patient encounters early in the curriculum, whereby the experience broadens and deepen the concepts learned in class and the post-session quiz provided opportunities for reflection and further conceptualization [25]. Additionally, the session incorporated elements of social theories of learning, focusing on social interactions, the patients as persons, and the spinal cord injury community. Gain of knowledge was demonstrated by their performance in the post-session quiz and the final exam. Our results support prior reports of enhanced learning outcomes associated with the incorporation of patient panels [24, 26]. It is possible that by recalling patients’ stories, students were able to make the appropriate connections and apply their knowledge to new patient scenarios in the assessments. By correctly identifying life-threatening situations on examinations, students could later apply these same concepts to real-life patients in the hospital setting. Noteworthy, student engagement and acquisition of knowledge may have been influenced by the incorporation of a graded quiz at the end of the session [25]. Although the performance of students on the final exam in a question regarding the identification of AD was above the national average, one question is not enough to make a strong conclusion.

In agreement with prior reports, we found that interacting with patients was associated with high learners’ satisfaction [24]. Most students considered that the session helped them understand SCI sequelae and its impact on patients. The highest level of satisfaction was regarding how well the MTP session helped students recognize AD and its triggers. This was not surprising to us since this was the core topic of the session, with more class time dedicated to it. In contrast, although spasticity was discussed and shown in class, there might have been difficult for all students to appreciate the demonstration in the large classroom, which may explain the lower satisfaction compared to AD. It is possible that this type of demonstration may be more meaningful if done within small groups. Given that the level of student satisfaction correlated with focus of the session, the time spent on each topic and questions prepared can be adjusted based on the specific learning objectives and goals of the session.

To our surprise, the level of satisfaction with the session for the second academic year was lower than the first year of implementation, despite no changes in knowledge acquisition. The major difference between both sessions was the number of patients, time of the session and student attendance (less in all counts for the class with lower satisfaction). There are some students in the second year who did not attend the session and yet filled out the satisfaction survey. Although our study design did not account for the reasons for these differences, one possibility is that some students not attending the session felt that they needed to answer the “satisfaction” questions since they were at the end of the graded quiz; answering the “satisfaction” questions without attending the session may have altered the data. On the other hand, there might be other differences between the sessions that may have accounted for the different levels of satisfaction. For example, there were discussions that happened in the first, but not the second year, including topics related to nutrition and foods that made bowel problems worse, sex life and orgasm as a trigger for AD, and the use of endocannabinoids for pain after SCI. These discussions incited a lot of interest in students and prompted them to participate more; this may have provided a greater holistic understanding of patients living with SCI and the impact of the disability on everyday life. In addition, one of the patients in the first session is a vocal advocate for people living with SCI and had ample public speaking experience, which may have been more impactful for the students.

Establishing a partnership between patients, faculty and students is essential to enhance the learning experiences of all participants [24, 27–29]. For our MTP session, we made a conscious effort to assure that our patients had a meaningful and rewarding encounter with students. Like prior reports, the primary role of our patient was “patient-teacher” and we purposely attempted to establish a partnership with patients where they felt involved and empowered [29] During the session planning, the patients were extensively briefed on the goals and audience, and they were empowered to suggest questions and topics for discussion. During the session, most patients felt comfortable using their experiences to participate in the teaching of basic elements of their condition, for example, about neurogenic bladder, catheterization, mechanism of action of the drug, etc. Emphasis was made on the proper communication language when interacting with people with disabilities. For example, patients gave student resources and tips during the session (e.g., avoid wheelchair bound, disabled person, handicapped, etc.).

Based on our experience, we recommend that all patients should be trained before the session and have at least one “rehearsal session”. Although advocates with public speaking experience might be preferred in some settings, other patients can be selected as long as they are invested in the learning process. Patients should not only be comfortable with the session format and content beforehand, but they should also be empowered to suggest and make changes that they believe are important to communicate with students. Furthermore, the session should incorporate opportunities that broaden students’ understanding of the condition beyond the concepts learned in class, such as the impact of the condition on everyday life. We believe it is important to provide opportunities for ample interactions between students and patients that help create positive connections and increase students comfort level when talking to people with disability. These observations are in agreement with prior reports in the literature [24]. Even though our session focused primarily on AD, the same principles can be applied to other conditions/diseases.

Our results have several limitations. We evaluated only short-term knowledge acquisition, we used a small number of questions, and there was no control group to evaluate the effectiveness of the MTP compared to other learning strategies. Although comparing pedagogies was not our objective, we cannot rule out that other methods might be as effective in helping students acquire the knowledge. Nonetheless, the MTP was originally designed to complement rather than substitute and may have benefits beyond imparting knowledge. This type of patient encounter may result in enhanced long-term retention, and/or changes in behavior or practice that can be transferred to patient care. This is an important question that merits more research, involving longer time points, adequate controls, and possibly more MTP sessions.

## Conclusion

Based on the high performance in knowledge-based questions and student satisfaction, we believe that the MTP session is an effective strategy to reinforce concepts related to SCI sequelae including AD and loss of bowel and bladder control. In addition to knowledge acquisition, the encounter with patients highlights to students the humanistic aspect of medicine and provides them with the opportunity to practice their communication skills. More research is needed to evaluate the long-term impact of these sessions.

The session can be adapted based on the specific learning objectives and needs of the course/program. For example, the session can be modified to cover other SCI complications such as increased risk for infections, coronary artery disease, serious abdominal complications, sexual dysfunction, etc., by changing the questions asked to patients or tasks given to the students. Naturally, this will require changing the preparatory materials and assessments. Moreover, MTP sessions can be used for any medical specialty and disciplines outside medicine. For example, patients can be invited to discuss side effects of drugs in a pharmacology class or family history to highlight mechanisms of inheritance in a genetics class. It is important when designing these sessions, to involve patients in the planning process, empowering them to make suggestions and take ownership of the learning experience.

## Data Availability

Survey data was provided by the Office of Medical Education at Herbert Wertheim College of Medicine. Quiz results were collected from CanvasMed Learning platform, and final exam performance was provided by the NBME assessment item analysis. All the information given to the authors is already reflected in the tables provided (no additional raw data to include). Questions are used for educational purposes and not for publication, but they can be provided by the corresponding author on reasonable request.
